# Expression of γH2AX in various gastric pathologies and its association with *Helicobacter pylori* infection

**DOI:** 10.3892/ol.2013.1693

**Published:** 2013-11-20

**Authors:** CHUAN XIE, LI-YAO XU, ZHEN YANG, XI-MEI CAO, WEI LI, NONG-HUA LU

**Affiliations:** Department of Gastroenterology, The First Affiliated Hospital, Nanchang University, Nanchang, Jiangxi 330006, P.R. China

**Keywords:** *Helicobacter pylori*, DNA double-strand breaks, γH2AX, gastric carcinogenesis

## Abstract

Phosphorylation of H2AX at Ser 139 (γH2AX) is a biomarker of DNA double-strand breaks (DSBs). The present study aimed to explore the association between γH2AX levels and gastric pathology and *Helicobacter pylori (H. pylori)* infection. Gastric biopsies were obtained from 302 *H. pylori*-negative and -positive patients, including those with chronic gastritis (CG), intestinal metaplasia (IM), dysplasia (Dys) and gastric cancer (GC). Proteins were extracted from five gastric epithelial cell lines and from 10 specimens of matched GC and adjacent normal tissues. The expression of γH2AX, a biomarker for the detection of DNA DSBs, in gastric tissues was detected by immunohistochemistry and western blotting. The expression of γH2AX progressively increased in tissues according to pathological stage from CG to Dys, but was slightly decreased in GC. *H. pylori* infection was associated with increased γH2AX expression, IM and Dys. Overexpression of γH2AX in GC was found to correlate with tumor location, gross appearance, differentiation, depth of invasion, TNM stage and lymph node metastasis. The results indicated that DSBs appear to be an early molecular event in gastric carcinogenesis, which may be associated with *H. pylori* infection. Moreover, immunohistochemical staining of γH2AX was found to correlate with a number of clinicopathological characteristics. The expression of γH2AX may serve as a valuable biomarker for the diagnosis and progression of GC.

## Introduction

Gastric cancer (GC) is the fourth most common malignancy and the second leading cause of cancer mortality worldwide (1). *Helicobacter pylori* (*H. pylori)* is a gram-negative bacteria that infects 50% of the global population. However, in certain regions and countries of the world, >80% of the population is infected with the bacteria. *H. pylori* has been defined by the International Agency for Research of Cancer as a class I carcinogen and is important for the progression from chronic superficial gastritis to chronic atrophic gastritis, intestinal metaplasia (IM), dysplasia (Dys) and finally GC (2).

DNA double-strand breaks (DSBs) are the most serious type of DNA damage and are frequently caused by ionizing radiation (IR), ultraviolet light and specific chemical agents. Recently, *H. pylori* has also been shown to induce DSBs in gastric epithelial cells *in vitro*(3,4). Inappropriate repair of DSBs can result in genomic instability, which has been shown to be a key factor in carcinogenesis (5). Phosphorylation of H2AX at Ser 139 (γH2AX) is abundant, fast, correlates well with DSBs and renders γH2AX a sensitive marker for the detection of DSBs (6). The expression of γH2AX has been shown to correlate with numerous types of malignant tumor and also with prognosis in early operable non-small cell lung cancer, vulvar squamous cell carcinoma and breast cancer (7–9).

There have been a number of studies on γH2AX expression in GC tissues. Sentani *et al*(10) showed that nuclear positive staining for GC was significantly higher than that in normal gastric tissues. However, no previous studies have investigated γH2AX expression in various gastric lesions or its correlation with *H. pylori* infection. Therefore, the aim of the present study was to measure the expression of γH2AX and determine its correlation with the various stages of gastric carcinogenesis, in the presence or absence of *H. pylori* infection.

## Patients and methods

### Patients and sample collection

Gastric tissue samples were collected from patients who had undergone an upper gastroduodenoscopy or gastrectomy at the First Affiliated Hospital of Nanchang University (Nanchang, China) between January 2007 and September 2008. A total of 302 patients ranging in age between 18 and 70 years were enrolled in the current study. The study included 56 cases of chronic gastritis (CG), 53 of IM, 47 of Dys and 146 of GC. None of the patients had been treated with proton pump inhibitors or antibiotics against *H. pylori,* and no GC patients had been treated with prior radio- or chemotherapy. The clinical characteristics of these patients are summarized in Table I. No significant differences were identified in the age or gender distribution among these groups. Clinicopathological characteristics were also obtained from the pathological reports.

In total, 10 GC tissue samples and adjacent normal tissues were collected from gastrectomy specimens at the First Affiliated Hospital of Nanchang University.

The present study was approved by the Ethics Committee of the First Affiliated Hospital of Nanchang University. All patients provided written informed consent prior to enrollment in the study.

### Histological examination

All biopsies or surgical specimens from the patients with gastric disease were obtained from the gastric antrum or lesion locations. The tissues used for histological analysis were fixed in 10% formaldehyde in Ca^2+^ and Mg^2+^-free phosphate-buffered saline (PBS) overnight at 4°C, prior to paraffin embedding. Paraffin sections, 4 μm thick, were sectioned with a microtome and stored at room temperature. Pathological diagnosis and classification were performed according to the criteria of the World Health Organization (11) and the updated Sydney system (12).

### Detection of H. pylori infection

Rapid urease test and modified Giemsa staining were used for the detection of *H. pylori* infection. The modified Giemsa staining was performed by two veteran pathologists. Consistency in the positive or negative results of the two tests was required.

### Immunohistochemistry

Slices were deparaffinized in dimethylbenzene, rehydrated through 100, 95 and 85% ethanol and incubated with fresh 3% H_2_O_2_ for 10 min to quench endogenous peroxidase activity. Microwave heating was used to expose antigens for detection. The primary antibody used for immunohistochemistry was rabbit monoclonal anti-human γH2AX (ab81299; 1:400; Abcam, Cambridge, UK). Slices were incubated at 4°C overnight and then washed with PBS three times. The secondary antibody (PV-6000; Zhongshan Golden Bridge Biotechnology Co., Ltd., Beijing, China) was incubated at 37°C for 30 min prior to reaction with 3,3-diaminobenzidine (Zhongshan Golden Bridge Biotechnology Co., Ltd.). Subsequently, slices were counterstained with hematoxylin and mounted with coverslips. Negative controls consisted of PBS without primary antibody (13).

### Review and scoring

The stained slices were reviewed and scored by two experienced pathologists. The concordance rates were generally high and results with any grading discrepancies were re-reviewed and discussed to determine a final score. Epithelial cells stained yellow or brown in the nuclei were defined as positive. Five fields for each slice were randomly selected, reviewed and scored (magnification, ×200). In each field, 100 immunoreactive cells were assessed and quantified as the percentage of total cells and then averaged from the five fields to calculate the percentage of immunostaining, i.e. 0, ≤5.0%; 1, 5.1–25.0%; 2, 25.1–50.0%; 3, 50.1–75.0%; and 4, >75.0%. Moreover, the staining intensity was also semi-quantitatively assessed as follows: 0, no staining; 1, weak staining; 2, moderate staining; and 3, strong staining. The integrals of the ‘area × intensity’ were calculated based on the following overall scores of the expression levels of the proteins in the sections: negative (−), 0–2; weak positive (+), 3–5; moderate positive (++), 6–8; and strong positive (+++), 9–12 (Table I) (13).

### Cell lines and culture

Five gastric mucosal cell lines were used in the present study, including GES-1 (immortalized gastric epithelial mucosa cell line, established by the Beijing Institute for Cancer Research, Beijing, China) and human gastric cancer SGC7901, MKN28, MKN45 and AGS cell lines (a gift from the Xijing Hospital of Digestive Disease, Xi’an, China). Cell lines were cultured at 37°C in an atmosphere of 5% CO_2_ in DMEM with 10% fetal bovine serum, 100 units penicillin and 100 μg/ml streptomycin (Gibco-BRL, Carlsbad, CA, USA) (14).

### Western blotting

Tissues and cells were lysed in a buffer containing 0.5% Lubrol-PX, 50 mM KCl, 2 mM CaCl_2_, 20% glycerol, 5 mM Tris-HCl (pH 7.4), 0.1% protease and 1% phosphatase inhibitors (Sigma-Aldrich, St. Louis, MO, USA). Following the addition of sodium dodecyl sulfate-polyacrylamide gel electrophoresis (SDS-PAGE) sample buffer, proteins were run on an SDS-PAGE gel and transferred to nitrocellulose membranes (Whatman GmbH, Dassel, Germany). The membranes were immunoblotted with antibodies against γH2AX (ab81299; 1:1,000; Abcam) and actin (1:1,000; Zhongshan Golden Bridge Biotechnology, Co., Ltd.). The reactions were subjected to incubation with an enhanced chemiluminescence detection system (Pierce Biotechnology, Inc., Rockford, IL, USA) and then exposed to X-ray film for visualizing the positive bands.

### Statistical analysis

SPSS 17.0 (SPSS Inc., Chicago, IL, USA) was used to perform the statistical analysis. Data are expressed as the mean ± standard deviation or percentage. The χ^2^ test was used to evaluate differences in categorical variables. The Kruskal-Wallis one-way analysis of variance and Mann-Whitney U tests were used to determine differences in numerical variables between various groups. P<0.05 was considered to indicate a statistically significant difference.

## Results

### Differential expression of γH2AX in various gastric lesions and its association with H. pylori infection

**Figure 1:**
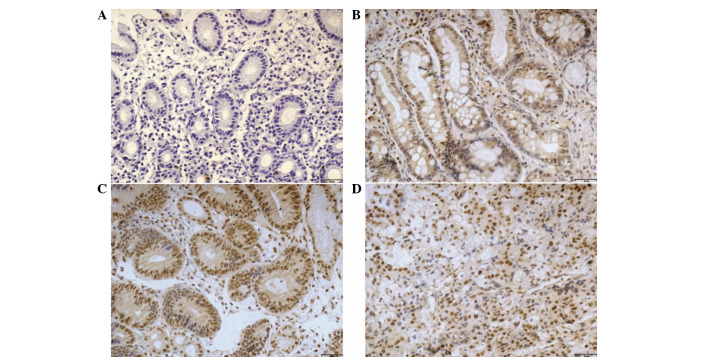
Immunohistochemical staining of γH2AX protein in (A) chronic gastritis, (B) intestinal metaplasia, (C) dysplasia and (D) gastric carcinoma tissues (magnification, ×200; scale bar, 40 μm). γH2AX, phosphorylated H2AX at Ser 139.

**Figure 2:**
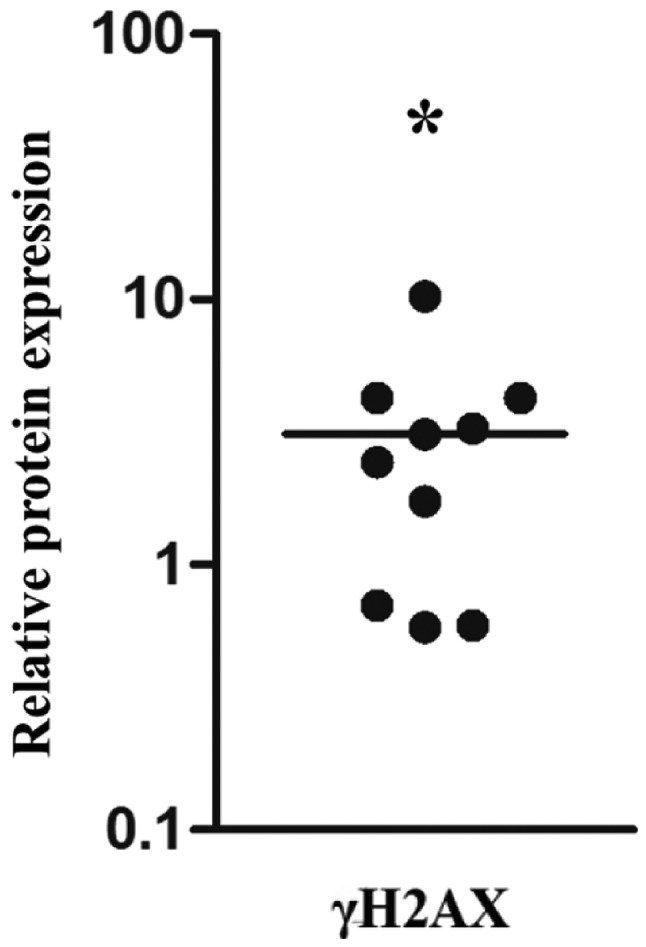
Relative γH2AX protein expression levels in gastric carcinoma compared with adjacent non-tumorous mucosa. Immunoblots of γH2AX were scanned and the relative protein expression level of tumor samples were compared with their adjacent non-cancerous counterparts and expressed as a percentage of β-actin. ^*^P<0.001. γH2AX, phosphorylated H2AX at Ser 139.

Immunohistochemical analysis showed that γH2AX was primarily found in the nuclei of epithelial cells. Semi-quantitative results of the expression of γH2AX are shown in Table I. The results showed that the expression ratio of γH2AX was 48.2% in the CG group, 73.5% in the IM group, 95.7% in the Dys group and 89.7% in the GC group. Consistent with the observations of Correa *et al*(2), the expression levels of γH2AX in the current study were significantly increased as pathological stages progressed from CG to Dys (P<0.001). However, expression levels were decreased in GC (P=0.011) (Fig. 1 and Table I). The expression of γH2AX was also measured by western blotting in GC and adjacent normal tissues. The results showed high γH2AX expression in GC tissues compared with adjacent normal tissues (P<0.001) (Fig. 2), which is consistent with the immunohistochemical results.

Moreover, in patients with IM and Dys, the expression of γH2AX was significantly higher in the presence of *H. pylori* infection (96.5 and 100%, respectively) compared with those without the infection (45.8 and 90.4%, respectively) (P=0.001 and P=0.008) (Table II). However, no significant differences were detected in the CG or GC groups.

The association of γH2AX expression with clinicopathological parameters was analyzed in 146 GC patients (Table III). The gastric body and cardia cancers showed a higher γH2AX expression (97.2%) than gastric antrum cancers (82.4%) (P<0.001). In addition, the expression of γH2AX in Borrmann III and IV type GC (90.5%) was significantly higher than Borrmann I and II type GC (88.9%) (P=0.002 ). γH2AX expression in poorly- and undifferentiated GC (93.8%) was significantly higher compared with that in well- and moderately differentiated GC (74.1%) (P<0.001). In cancer tissues located in the submucosa, the γH2AX expression (85.1%) was significantly lower than that in cancer tissues that had reached the subserosal level (91.9%) (P=0.002). Furthermore, γH2AX was expressed more frequently in TNM III and IV stage patients (91.9%) than in TNM I and II stage patients (86.4%) (P<0.001), and there was higher γH2AX expression in the patients with lymph node metastasis (91.2%) than in the patients without (84.8%) (P=0.002).

### Differential expression of γH2AX in various gastric epithelial cell lines

**Figure 3:**
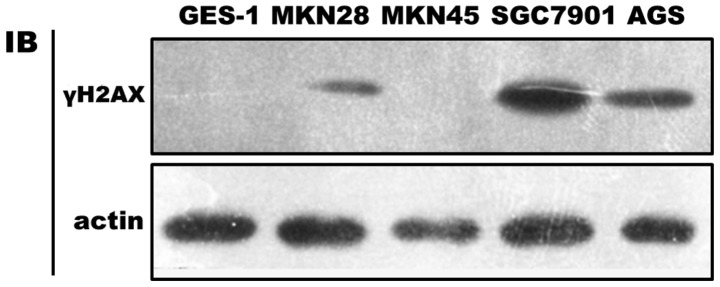
Expression of γH2AX in non-cancerous and cancerous gastric cell lines measured by western blotting. Cell lines were grown for two days and then subjected to total cellular protein isolation. γH2AX, phosphorylated H2AX at Ser 139.

Expression of γH2AX was higher in GC cell lines (i.e., SGC-7901, MKN-28 and AGS, with the exception of MKN-45) compared with the non-cancerous cell line GES-1 (Fig. 3).

## Discussion

DSBs are important threats to genome integrity causing chromosomal aberrations, which are remarkable characteristics of malignant tumors (15). While the response to DNA damage acts as an anticancer barrier in early human tumorigenesis, γH2AX has been reported to be highly overexpressed in precancerous lesions of the urinary bladder, breast, lung and colon cancer (16). In the current study, the expression of γH2AX was shown to progressively increase in accordance with the pathological progression from CG to Dys. γH2AX expression in GC was also higher than that in CG, but was slightly decreased in Dys. The reason for lower γH2AX expression in GC compared with Dys remains unclear; however, restoration of genomic instability during tumor progression may explain this observation (17,18). The western blotting results of the cancer and adjacent normal tissues were confirmed by cell line experiments. All results showed that the response to DNA damage was activated in gastric precancerous lesions.

Recently, Toller *et al* have shown that *H. pylori* triggers DSBs in gastric epithelial cells in cell culture, primarily mediated by *H. pylori* blood group antigen-binding adhesion (3). In addition, Jang *et al* have shown that *H. pylori*-induced DSBs may be inhibited by lycopene (4). The two investigations were based on AGS coculture with *H. pylori* in cell culture, a model system which is different from the clinical situation. The results of the current study were based on human samples and showed that high expression of γH2AX in IM and Dys patients appeared to correlate with *H. pylori* infection, which implied that *H. pylori* not only induces DSBs in cell culture, but may also be involved in an early molecular event in gastric tumorigenesis. However, whether the DNA damage response pathways are impaired requires further study.

The observations of the current study have shown that the overexpression of γH2AX in GC correlates with a number of clinicopathological characteristics, including tumor location, tumor gross type, differentiation, tumor invasion depth, TNM stage and lymph node metastasis. All these correlations implied that, as the degree of malignancy increased, the genomic instability was also significantly increased. These results confirm previous observations by Sentani *et al*(10), in which the expression of the γH2AX protein in GC was significantly higher in non-neoplastic gastric mucosa and its high expression was also found to correlate with stage II–IV cases. Exposure to IR was an important factor in the study.

In conclusion, DSBs appear to be an early molecular event in gastric carcinogenesis, which is associated with *H. pylori* infection and a number of clinical characteristics. Although the exact mechanism by which DSBs are induced by *H. pylori* infection remains unclear, the expression of γH2AX may serve as a valuable biomarker for the diagnosis and progression of GC.

## Figures and Tables

**Table I:** Expression of γH2AX in patients with various histological observations.

				Overall score of γH2AX expression					
				
Group	n	Mean age (SD), years	Gender, M/F	−, n	+, n	++, n	+++, n	%^a^	P-value
1 CG	56	53.6 (10.7)	30/26	29	27	0	0	48.2	
2 IM	53	54.3 (9.6)	29/24	14	26	12	1	73.5	<0.001^b^
3 Dys	47	55.1 (10.3)	26/21	2	9	27	9	95.7	<0.001^b^,^c^
4 GC	146	56.8 (14.1)	96/50	15	57	50	24	89.7	<0.001^b^; 0.011^d^

a Percentage of immunostaining.

b vs. CG group;

c vs. IM group;

d vs. Dys group.

γH2AX, phosphorylated H2AX at Ser 139; M, male; F, female; CG, chronic gastritis; IM, intestinal metaplasia; Dys, dysplasia; GC, gastric cancer.

**Table II:** Expression of γH2AX in patients with various histological observations, in relation to *H. pylori* infection.

					Overall score of γH2AX expression					
					
Group	*H. pylori*	n	Mean age (SD), years	Gender, M/F	−, n	+, n	++, n	+++, n	%^a^	P-value
CG	+	26	54.1 (11.7)	14/12	7	19	0	0	73.1	0.362
	−	30	53.2 (9.1)	16/14	22	8	0	0	26.7	
IM	+	29	54.8 (8.8)	14/15	1	19	8	1	96.5	0.001
	−	24	53.7 (10.3)	15/9	13	7	4	0	45.8	
Dys	+	26	54.3 (12.2)	13/13	0	2	17	7	100.0	0.008
	−	21	56.1 (9.5)	13/8	2	7	10	2	90.4	
GC	+	61	58.3 (16.3)	39/22	6	25	20	10	90.2	0.865
	−	85	50.8 (13.9)	57/28	9	32	30	14	89.4	

a Percentage of immunostaining.

γH2AX, phosphorylated H2AX at Ser 139; *H. pylori: Helicobacter pylori;* M, male; F, female; CG, chronic gastritis; IM, intestinal metaplasia; Dys, dysplasia; GC, gastric cancer.

**Table III:** Clinicopathological association of γH2AX expression in patients with GC.

		Overall score of γH2AX expression					
		
Characteristics	n	−, n	+, n	++, n	+++, n	PR, %	P-value
Gender							
Male	96	8	39	35	14	91.7	0.923
Female	50	7	18	15	10	86.0	
Age, years							
≥55	86	7	36	28	15	91.8	0.780
<55	60	8	21	22	9	86.7	
Location							
Antrum	74	13	31	25	5	82.4	<0.001
Body and cardia	72	2	26	25	19	97.2	
Gross type (Borrmann)							
I and II	72	8	37	21	6	88.9	0.002
III and IV	74	7	20	29	18	90.5	
Differentiation							
Well and moderately	81	11	40	21	9	74.1	
Poorly and undifferentiated	65	4	17	29	15	93.8	<0.001
Invasive depth							
Above submucosa	27	4	18	3	2	85.1	
Muscularis propria	8	2	0	3	3	75.0	0.080^a^
Below subserosa	111	9	39	44	19	91.9	0.002^a^; 0.373^b^
TNM							
I and II	59	8	35	12	3	86.4	<0.001
III and IV	87	7	22	38	20	91.9	
Lymph node metastasis							
With	113	10	37	45	21	91.2	0.002
Without	33	5	20	5	3	84.8	

a vs. above submucosa;

b vs. muscularis propria.

γH2AX, phosphorylated H2AX at Ser 139; GC, gastric cancer; PR, positive rate.
